# Immune Transcriptome of Cells Infected with Enterovirus Strains Obtained from Cases of Autoimmune Thyroid Disease

**DOI:** 10.3390/microorganisms9040876

**Published:** 2021-04-19

**Authors:** Anello Marcello Poma, Sarah Salehi Hammerstad, Angelo Genoni, Alessio Basolo, Knut Dahl-Jorgensen, Antonio Toniolo

**Affiliations:** 1 Department of Surgical, Medical, Molecular Pathology and Clinical Area, University of Pisa, 56126 Pisa, Italy; 2 Department of Pediatric Medicine, Oslo University Hospital, 0450 Oslo, Norway; hammerstad.sara@gmail.com (S.S.H.); knut.dahl-jorgensen@medisin.uio.no (K.D.-J.); 3 Specialist Center Pilestredet Park, Pilestredet Park 12.A, 0176 Oslo, Norway; 4 Medical Microbiology, Department of Biotechnology and Life Sciences, University of Insubria, 21100 Varese, Italy; angelopaolo.genoni@uninsubria.it; 5 Department of Clinical and Experimental Medicine, University of Pisa, 56126 Pisa, Italy; alessio.basolo@med.unipi.it; 6 Faculty of Medicine, The University of Oslo, 0316 Oslo, Norway; 7 Global Virus Network, University of Insubria, 21100 Varese, Italy; antonio.toniolo@gmail.com

**Keywords:** thyroiditis, autoimmunity, virus, persistent infection, pathogenesis, interferon, cytokine, CCL2, IL-18

## Abstract

Background: Hashimoto’s thyroiditis and Graves’ disease are autoimmune thyroid disorders (AITD) of unknown origin. Enterovirus (EV) infection of thyroid cells has been implicated as a possible initiator of cell damage and of organ-specific autoimmunity. We asked whether persistent infection of human epithelial cells with EV strains obtained from thyroid tissue of AITD patients could be associated with transcriptional changes capable of fostering immunopathology. Methods: EV isolates obtained from thyroid tissue of AITD cases were used to infect the AV3 epithelial cell line. AV3 cells incubated with a virus-free medium from thyroid tissue of subjects without evidence of thyroid autoimmunity were used as uninfected controls. Transcripts of immune-related genes were compared in infected vs. uninfected cells. Results: The EV genome and antigens were detected only in the cells exposed to AITD-derived virus isolates, not in control cells. Persistent EV infection, while suppressing transcription of several type I IFN and cytokine determinants, was associated with enhanced transcription of NFKB1/RELA, IFNAR1, JAK1/STAT1, i.e., the determinants that play key immunologic roles. Infection also led to upregulation of the CCL2 chemokine and the IL-18 pro-inflammatory interleukin. Conclusion: As in the case of EV strains obtained from autoimmune diabetes, results show that the EV strains that are present in the thyroid of AITD cases do repress IFN and cytokine pathways. JAK1/STAT1 upregulation supports activation of TLR pathways and aberrant T cell signaling. In the early phases of AITD, our results highlight the potential benefit of interventions aimed at blocking the viral infection and easing the inflammatory response.

## 1. Introduction

Autoimmune thyroid disorders (AITD) are linked with immune-mediated insults to the follicular epithelial cells that produce the T3 and T4 hormones. Hashimoto’s thyroiditis (HT) is the most prevalent cause of hypothyroidism via progressive damage of thyrocytes [1]. Graves’ disease (GD) is also common and causes hyperthyroidism due to excessive production of thyroid hormones via stimulatory autoantibodies directed to the thyrotropin receptor [2]. In both cases, undetermined stimuli are deemed to break immune tolerance and promote organ-specific autoimmunity [3].

Genome-wide association studies (GWAS) revolutionized the field of AITD genetics by enabling the detection of several susceptibility loci [4], including the HLA class I region, *FCRL3* and *TSHR* genes as well as immune-related genes [3] such as *CTLA4* (encoding a protein involved in immune checkpoints), *CD25* and *CD40* (encoding proteins that regulate T cell activity), *PTPN22* (encoding a protein involved in T cell signaling) and *FOXP3* (encoding a transcription factor involved in the development of regulatory T cells that modulate the immune response).

As individual genes provide a small part of the calculated genetic risk, the interaction of the genes with each other and with non-genetic factors is important [2]. In the onset and progression of AITD, susceptibility loci support a role both for aberrant T cell and B cell signaling and for environmental determinants of thyroid disease.

Several studies indicate that a chronic low-grade viral infection of target organs may represent the initial stimulus for autoimmune conditions, including multiple sclerosis [5], type 1 diabetes mellitus (T1D) [6,7] and AITD [8].

While an acute infection causes cell death and is usually symptomatic, a persistent low-grade infection is subclinical and may excite prolonged responses in infected cells that may gradually activate immune pathways [9].

In T1D, we demonstrated a low-grade persistent enterovirus (EV) infection of the pancreatic islets of Langerhans [10,11]. By immunostaining, the expression of the EV capsid protein VP1 was detected in the pancreas of up to 80% T1D donors in the nPOD cohort [7]. Other studies of T1D patients have shown that EV genomes and infectivity are present in peripheral blood leukocytes, spleen and lymph nodes [12,13]. In pancreatic islets, the demonstrated upregulation of HLA class I and *STAT1* genes likely reflects an enduring antiviral response [14]. In fact, transcriptome analysis of the cells infected with EV strains from T1D cases show that distinct immune pathways are deregulated [9].

The association of AITD with microbial infections has long been studied [8,15]. Our group investigated AITD pathogenesis obtaining results reminiscent of those found in T1D. The EV capsid antigen has been detected more frequently in thyrocytes of GD patients than in controls [16], EV genomes have been frequently demonstrated in AITD patients, poorly replicating enteroviral isolates have been obtained (AT and KD-J; MS in preparation).

To investigate the effects of the EV strains derived from AITD cases on human epithelial cells, the immortalized AV3 cell line was infected with the above strains and the expression of immune-related genes was assessed. Non-infected AV3 cells were used for comparison. The results indicate that infection by AITD-derived EV strains curbs antiviral defenses and activates pathways potentially capable of initiating organ-specific autoimmunity [9,16].

## 2. Materials and Methods

Instrumentation, consumables, cell lines, culture media, chemicals, molecular biology reagents, antibodies, commercial kits, software and applications are listed in Supplementary Table S1.

### 2.1. Human Samples

Frozen thyroid tissue samples obtained from 53 female patients were collected for immunologic and virology studies [17]. Tissues from AITD patients and controls with no evidence of thyroid autoimmunity were taken at the Oslo University Hospital (age range, 38–69 years). Control samples were obtained from patients undergoing neck surgery for primary hyperthyroidism. The samples were snap-frozen in liquid nitrogen and stored at −80 °C. To assess pre-existing or unrecognized thyroid autoimmunity, serum thyroid hormones and thyroid autoantibodies were measured. The following methods were employed: TSH and FT4 (DELFIA sandwich immunoassays, Perkin Elmer, Turku, Finland), FT3 (immunoenzymatic assay, Siemens Healthcare, Erlangen, Germany); TR-Ab (competitive immunoassay; BRAHMS GmbH, Berlin, Germany), TPO-Ab and Tg-Ab (sequential immunoassay; Immulite, Siemens, Los Angeles, CA, USA). For virus studies, the samples were shipped in dry ice to a virology laboratory. The observational study was approved by the Ethics Committee of Ospedale di Circolo and Fondazione Macchi (Varese, Italy; #2018/17233, approved on 30 October 2018) and was performed in accordance with the Declaration of Helsinki and local regulatory laws. Once thawed, aliquots of each sample were used for virus detection. Residual amounts were suspended in fetal bovine serum plus 10% DMSO, frozen gradually and stored in liquid nitrogen for further use. For transcriptome analysis, eight thyroid samples were selected from the above collection of 53 samples (four EV-positive AITD cases and four cases with no evidence of thyroid autoimmunity).

### 2.2. Cells and Virus Strains

Following a published method [9], a mixture of five EV-permissive cell lines that express a wide variety of EV receptors was used for virus isolation. The AV3, RD, 1.1B4, VC3 and HEK293 cell lines (obtained from the European Collection of Authenticated Cell Cultures, ECACC) were cultured at 37 °C in air with 5% CO_2_ using the DMEM/F12 medium supplemented with L-glutamine, heat-inactivated 10% fetal bovine serum (FBS), penicillin/streptomycin. Cell cultures were checked monthly for mycoplasma contamination (MycoAlert Plus Mycoplasma kit, Euroclone-Lonza, Pero, Italy).

Transcriptome analysis was performed using a single cell line (AV3 cells) since (1) the preliminary transcriptome analysis of the above mixture of five different cell lines produced poorly reproducible results; (2) the AV3 cell line is known to be susceptible to many different EV types; (3) the transcriptome analysis of AV3 cells alone provided reproducible results.

The transcriptome analysis of AV3 cells acutely infected by coxsackievirus B3 (CBV3, Nancy strain) has been already published by our group [9]. Thus, in this study, the CBV3 was also used to set up the experimental system. CBV3 is capable of infecting thyrocytes [16]. The results of acutely infected AV3 cells were superimposable to those already published [9].

For acute infection, confluent cultures in T25 flasks were incubated (rocking platform at 37 °C) with 1 mL of the diluted virus (multiplicity of infection = 0.1). After 1 h, cell monolayers were washed with a warm medium, and the flasks were refilled with 5 mL medium. The cultures were then incubated at 37 °C for 72 h. For persistent infection, AV3 cells were incubated with undiluted EV isolates derived from AITD cases, treated as above and incubated at 37 °C for 72 h before harvesting.

### 2.3. EV Detection in Thyroid Tissue Following Enrichment in Cell Culture

A published procedure for isolating and detecting EV strains causing persistent infection was followed [18]. Briefly, homogenates of thyroid tissue were co-cultured with a mix of EV-susceptible cell lines (AV3, RD, 1.1B4, VC3, HEK293) in order to enrich for virus (2–3 passages). For each sample, RNA was extracted from two T25 flasks (supernatant plus cells), then reverse-transcribed (RT) using the Superscript III reverse transcriptase and the VILO master mix (ThermoFisher, Waltham, MA, USA). Four different EV-specific RT-PCR end-point assays were performed [18]. In addition, RT-PCR assays for the region encoding the capsid protein VP1 were carried out according to published protocols [19,20]. A LabChip GX Touch 24 analyzer (Perkin Elmer Italia, Milan, Italy) based on capillary electrophoresis was used to detect amplicons. A PCR test was deemed positive when an amplicon peak of the expected size was observed in the electropherogram and its viral nature was confirmed by Sanger sequencing. EV-negative and EV-positive supernatants of AV3 cell cultures were stored at −70 °C and used for subsequent infection experiments.

### 2.4. Detection of EV Antigens in Infected AV3 Cells

Cell monolayers were prepared in Millicell EZ 4-well glass slides (Merck, Darmstadt, Germany) and fixed in PBS containing 4% paraformaldehyde. Expression of EV protein antigens was evaluated by immunofluorescence with anti-EV monoclonal antibodies (Table S1) targeting either the VP1 capsid protein (mAbs 9D5, 6-E9/2, 5D-8.1) [21] or the viral 3D RNA polymerase (mAb 3D-02 and 3D-05 from our own laboratory). For additional virus typing, select monolayers were also stained with mAbs specific for group B coxsackieviruses; echoviruses 4, 6, 9, 11, 30, 34; polioviruses 1–3 (Table S1). Alexa Fluor 488-goat anti-mouse IgG (ThermoFisher) was used as secondary antibody. The slides were counterstained with Blue Evans. VP1 staining was deemed positive if fine granular cytoplasmic fluorescence was detected in infected cells. In persistently infected cells, the 3Dpol staining typically produced diffuse speckled fluorescence in the nuclear area. The images were taken with a Nikon E80i microscope and adjusted in brightness and contrast using Adobe Photoshop.

### 2.5. Immune Transcriptome of Uninfected and Infected AV3 Cells

For each sample, total RNA was isolated with the RNeasy Mini Kit (Qiagen, Hilden, Germany) from two T25 flasks containing either infected cells or uninfected control cells. RNA quality was assessed by spectrophotometry (Trinean, Gentbrugge, Belgium). Gene expression assays were carried out as reported [9] using the nCounter Human Immunology v2 Panel (nanoString Technologies, Seattle, WA, USA).

### 2.6. Statistical Analysis

For the statistical analysis, experimental groups were compared using either the unpaired two-tailed Student’s *t*-test or the Mann–Whitney test (GraphPad Prism v.8.0.0 for Windows, GraphPad Software, San Diego, CA, United States, www.graphpad.com, last accessed 22 December 2020). The *p*-value < 0.05 was considered statistically significant.

For the transcriptome analysis, normalized counts were obtained by background thresholding as reported [22] by using the maximum value of negative control counts as the threshold. Patterns of gene expression were assessed by unsupervised hierarchical clustering using Euclidean distance and Ward as distance and clustering methods, respectively. The Bayes moderated *t*-statistics was used to compute differentially expressed genes (DEG); a false discovery rate (FDR) below 0.05 was considered significant. The gene set analysis was performed by the procedures of gage v.2.34.0. All the analyses were performed in the R environment (version 4.0.2, https://www.r-project.org/, last accessed 22 December 2020) unless otherwise specified.

## 3. Results

Female subjects from 38 to 69 years of age were studied (Table 1). Thyroid tissues of AITD cases (*n* = 4) were investigated in comparison to cases with no evidence of thyroid autoimmunity (*n* = 4). GD cases were characterized by low TSH levels and somewhat enhanced FT3 or FT4 levels together with positivity for thyroid receptor-stimulating autoantibodies (TR-Ab), reduced levels of serum TSH and possible positivity for other thyroid autoantibodies. HT was characterized by high TSH levels, possibly low FT3 and FT4 levels and positivity for one or more thyroid autoantibodies.

Repeated experiments showed that incubation of thyroid tissue with EV-susceptible cell cultures allowed isolation of EV strains from the four selected AITD cases, not from the four selected cases that had no evidence of thyroid autoimmunity. As previously published [18], prolonged observation using time-lapse microscopy of infected cell cultures suggested EV positivity in the form of a slowly developing cytopathic effect (images taken every 30 min for 70–200 h). The cytopathic effect could be reproduced upon serial passage. EV positivity was proven directly by detecting the EV genome/protein in cultures using both RT-PCR and immunostaining. Amplicon size and sequence were determined by capillary electrophoresis and Sanger sequencing, respectively. Table 2 shows short genome sequences of the EV isolates obtained from four AITD cases. Sequences refer to the untranslated 5′ region of the EV genome, a region that is more easily detected when dealing with extremely tiny viral loads. Comparison of the sequences to those present in public databases indicates that the isolates belong to the enteroviral A, B species or to the rhinovirus C species.

**Figure 1:**
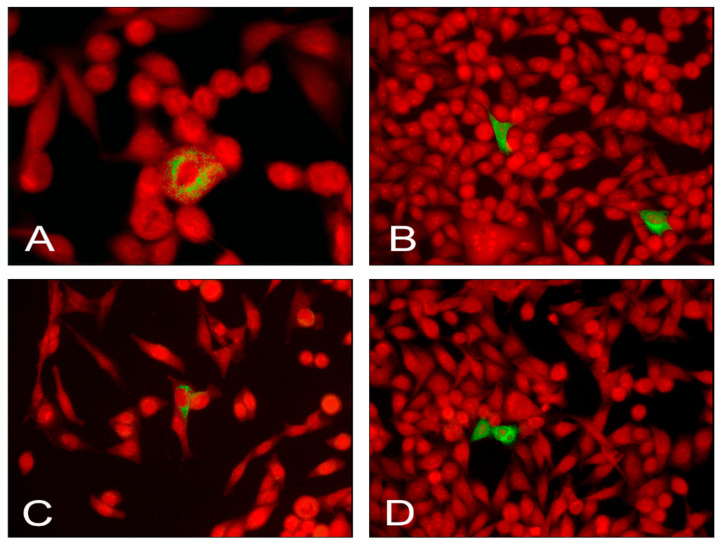
Green immunofluorescent staining of the enteroviral capsid protein VP1 (MAb 6-E9/2) in the AV3 cell line infected with persistent enterovirus (EV) strains isolated from AITD cases. Red, Evans Blue counterstaining. EV strains from cases T21 (**A**), T23 (**B**), T148 (**C**), T131 (**D**). Original magnification: 40×, A; 20×, B, C, D.

Since determination of EV genotypes requires sequencing of the viral capsid-coding region, we attempted to amplify the VP1-coding tract [19,20]. Yet, our efforts were not successful, presumably due to the extremely low virus loads combined with the huge variability of the VP1-coding region between the numerous virus types belonging to the *Enterovirus* genus and the possible inadequacy of the employed primers (Enterovirus, http://Picornaviridae.com/, accessed on 5 November 2019). Though only tiny amounts of virus were present, the VP1 enteroviral protein could be detected in infected cells. Positivity was demonstrated using three distinct antiviral antibodies. Figure 1 shows the cytoplasmic fluorescence produced by the VP1 antibody 6-E9/2 in cases T21, T23, T131, T148. Weaker fluorescent staining was also obtained with antibodies to the enteroviral 3D RNA-dependent RNA polymerase (MAbs 3D-02 and 3D-05; not shown). As expected, enteroviral antibodies did not stain AV3 cells that had been incubated with virus-free thyroid tissue of subjects with no evidence of autoimmunity.

In summary, the viral isolates obtained from AITD cases were attributed to the *EV* genus based on partial sequencing of the 5′UTR genome region and on the immunodetection of the enteroviral VP1 and 3Dpol antigens using independent antibodies.

To characterize the transcriptome of infected vs. noninfected AV3 cells, RNA transcripts of 580 immune-related genes were assessed in AV3 cells that had been incubated with either a medium containing virus strains of AITD cases or a virus-free medium from the cells incubated with tissue of controls showing no evidence of thyroid autoimmunity.

**Figure 2:**
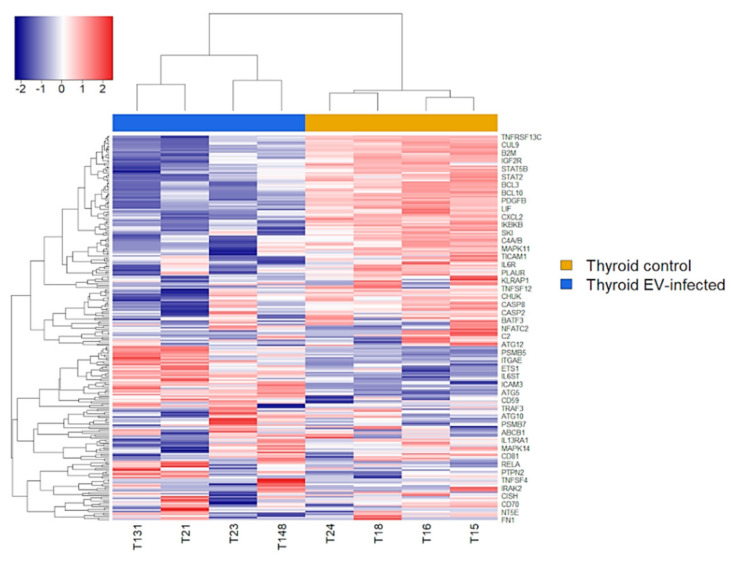
Unsupervised hierarchical clustering of differentially expressed genes. EV-infected cells vs. uninfected cells (left four lanes and right four lanes, respectively). Samples and genes were independently clustered based on the gene expression profile. In the heatmap, red and blue represent high and low expression levels, respectively.

After normalization and filtering out of low count targets, a total of 241 genes were considered. Hierarchical clustering produced a clear distinct transcription profile between EV-infected and control samples, with a predominance of gene downregulation in EV-positive samples (Figure 2).

**Figure 3:**
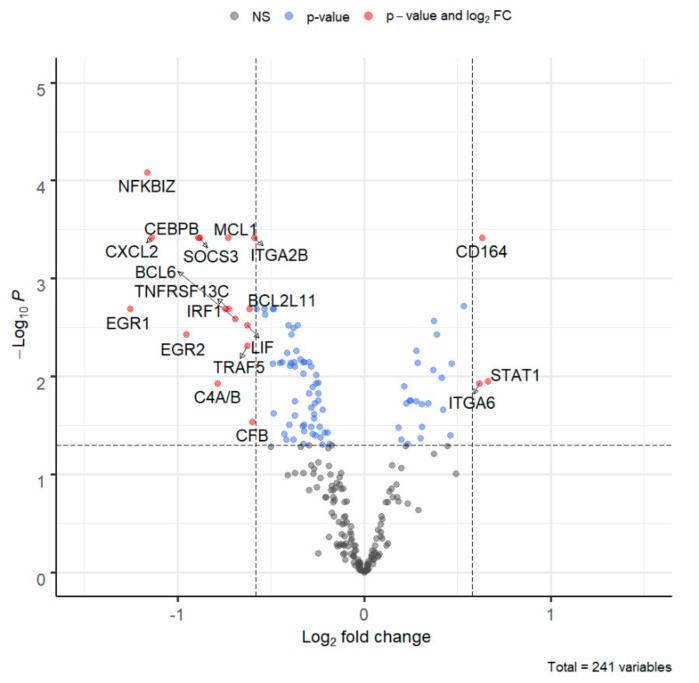
Volcano plot. Log_2_ fold change and −log_10_ of the adjusted *p*-value of the differential expression analysis were plotted. The horizontal line represents the adjusted *p*-value of 0.05. Vertical lines correspond to −0.58 and 0.58 log_2_ fold changes, which are equivalent to the −1.5 and 1.5 linear fold changes, respectively. Blue dots are differentially expressed genes; red dots are the genes differentially expressed with an absolute value of log_2_ fold change greater than 0.58.

The results were confirmed using the DEG analysis (Table S2) as seen in the volcano plot (Figure 3). Compared to uninfected AV3 cells, EV infection is associated with reduced expression of multiple genes, including *CFB*, *TRAF5*, *LIF*, *SOCS3*, *CXCL2*. The downregulated genes are mainly determinants of innate immunity. On the right side of the volcano plot are a few upregulated genes, notably *ITGA6* (an integrin), *CD164* (an adhesion molecule that may also act as an antiviral factor) and *STAT1* (a transcription activator that mediates the response to interferons).

Analysis of type I interferon (IFN) pathways in infected vs. uninfected cells (Table 3) showed downregulation of 12/25 genes in the IFN induction pathway and of 3/8 genes in the IFN signaling pathway. Notably, persistent infection upregulates transcription of *NFKB1* and *RELA* genes in the IFN induction pathway, as well as of *IFNAR1*, *JAK1* and *STAT1* in the IFN signaling pathway. The NFKB1–RELA complex is a transcriptional activator of multiple genes, including type I IFNs. IFNAR1 is a component of the receptor for type I IFNs. Upon binding with exogenous IFN, the receptor activates the JAK–STAT signaling cascade that mediates the cellular response to IFNs and cytokines.

Analysis of cytokine-related transcripts in infected vs. uninfected cells also revealed a pattern of predominant downregulation (Table 4). First, transcription of regulatory genes of the inflammatory process and those preventing excessive immune responses was repressed (*EGR1*, *EGR2*, *CEBPB*, *SOCS3*); second, transcription of multiple cytokine genes was also repressed (*IL1B*, *IL6*, *CXCL2*, *LIF*, *IL20*, *TGFB1*, *PDGFB*, *GP*I). Notably, two genes were upregulated: the *CCL2* chemokine (a chemoattractant for myeloid and lymphoid cells) and *IL18* (a proinflammatory interleukin that is involved in autoimmune processes).

Bioinformatic analysis showed that three signaling pathways were activated by persistent infection: the neurotrophin, the T cell receptor and the toll-like receptor (TLR) pathways (Table 5). Triggering appears to be driven by the transcription of genes that act mainly downstream the signaling cascade. Upregulation of *RAF1* and *NFKB1* plays a role in activating both the neurotrophin and the T cell receptor signaling pathways. Upregulation of *IFNAR1*, *NFKB1*, *STAT1* contributes to activating the TLR signaling pathway.

## 4. Discussion

When a microbe escapes eradication and succeeds in establishing a persistent infection, it initially causes minor host damage without producing clinical symptoms [23]. Persistence has advantages for both the host and the microbe but may herald slowly progressing diseases such as cancer and autoimmunity [24,25,26,27].

In the context of autoimmune diabetes, pancreatic islets infected in vitro with echovirus strains isolated in the course of Cuba epidemics showed upregulated transcription of TLR3 and IFN-beta genes [28]. During the abovementioned epidemics, some children were infected by echovirus. Successively, part of them seroconverted to diabetes-related autoantibodies or progressed directly to autoimmune diabetes. Thus, in vitro studies of virally infected islets support the contention that pancreatic autoimmunity derives from the ability of particular viral strains to modulate gene expression in islet cells and to activate innate immunity factors implicated in organ-specific autoimmunity [29].

The same seems to hold in the context of AITD. Development of HT and GD is particularly frequent in subjects chronically infected with hepatitis C virus (HCV). In cultured thyroid cells, HCV promotes the transcription of IL-6, IL-8 and heat shock proteins [30]. The effect is mediated, in part, by the envelope-2 (E2) protein of the virus that interacts with PKR and blocks its inhibitory effect on protein synthesis and cell growth [31]. The interaction of E2 and PKR is one mechanism by which HCV circumvents the antiviral effect of interferon.

Currently, multiple viral pathogens are regarded as possible triggers of AITD, including HCV and EV. It should be noted that, as a common factor, all of them share the ability to activate the innate response in thyrocytes [32].

Disruption of protein homeostasis is a key contributor to the pathogenesis of many human disorders, including those of viral and prion etiology [33]. The autophagy-mediated recycling of misfolded proteins and aggregates serves as a key component of protein quality control and preserves physiological functions. Modified host proteins are present in EV-infected cells. For instance, coxsackievirus B3 infection causes the proteolytic cleavage and loss-of-function of autophagy adapter proteins SQSTM1 and NBR leading to accumulation of ubiquitin conjugates and cell damage [34]. EVs express two main proteases (2A and 3C). During EV infection, the protein LSM14A (essential for the formation of processing bodies, structures involved in mRNA splicing) is cleaved, thus disrupting its multiple antiviral functions [35]. The 2A protease of EV-D68 inhibits type I IFN responses by cleaving tumor necrosis factor receptor-associated factor 3 (TRAF3) which is a key factor for producing type I IFN [36]. Structural enteroviral proteins such as VP1 may also participate in suppressing the antiviral state in infected cells [37]. Finally, frequent and widespread alternative splicing events have been documented in cells infected with EV-71 [38] and profound changes of the proteome occur in human cells infected with coxsackievirus B3 [39].

Consequently, it is reasonable to assume that persistently infected cells also undergo similar events and produce cleaved, misfolded and ubiquitinated protein antigens that are continuously presented to the immune system. Mechanisms of this kind may well contribute to the genesis of EV-associated autoimmunity.

Our findings show that the thyroid-derived EV strains causing persistent infection of AV3 cells are associated with (a) a slowly-developing cytopathic effect possibly associated with the production of incomplete non-encapsidated viral particles [40], (b) widespread downregulation of type I IFN induction and signaling genes, (c) upregulation of multifunctional genes (*NFKB1*/*RELA*, *JAK1*/*STAT1*, *IFNAR1*), (d) downregulation of cytokine and transcription regulators involved in inflammatory processes, (e) upregulation of the chemotactic and pro-inflammatory factors *CCL2* and *IL18*. The above changes reduce the innate antiviral response that is instead promoted by acute infection [9], favor virus persistence, stimulate the recruitment of inflammatory lymphoid cells and foster inflammatory changes. Thus, anti-cytokine agents and/or JAK1 inhibitors could be considered for therapeutic interventions in the early phases of AITD [7]. In this study, the expression of transcripts was not validated by quantitative analysis of protein expression. Evaluation of protein products playing a role in IFN induction and signaling is the aim of ongoing research using a wider collection of thyroid samples.

Our in vitro model, though capable of sustaining a persistent EV infection and disclosing transcriptome changes, is inadequate to reproduce the complex pathogenic process leading to AITD. First—for simplicity—instead of primary thyroid epithelial cells, a reporter epithelial cell line that lacks endocrine differentiation was used. Second, unlike in vivo, in vitro, the infected cells are not exposed to the infiltrating inflammatory cells with their manifold effects. Third, for obvious reasons, the observation period had to be limited to a few days vs. the long time that is required in vivo to generate autoimmune reactions upon natural virus infection [25].

However—with these and other limits—persistently infected AV3 cells manifest transcriptome changes that mostly affect IFN pathways [41] and innate immunity. The results are thus analogous to those that were obtained with AV3 cells persistently infected by EV strains derived from the pancreas of T1D organ donors [9].

In closing, multiple hints suggest that exposure to EV strains causing persistent infection of endocrine cells (that are characterized by peculiar constituents not shared by other bodily cells) may incite autoimmune responses to self-constituents. Hence, viral infection may be an important environmental trigger for endocrine autoimmunity.

One crucial question in this context is whether the disease process would continue if persistent infection could be terminated. Indeed, a Norwegian trial with antiviral drugs in T1D is currently ongoing (DiViD intervention trial: EudraCT number 2015-003350-41) and a similar trial in AITD is being launched. The results of these trials might change the therapeutic strategies for immune-mediated endocrine diseases.

It has long been proposed that EV infections may trigger T1D in susceptible individuals. Currently, a polyvalent coxsackievirus B vaccine is being developed for reducing T1D incidence in children [42]. With improvements of virus detection methods and the possible implementation of antiviral drugs and vaccines, AITD will also likely benefit from antiviral interventions.

## Figures and Tables

**Table 1:** Clinical data of AITD cases selected for analysis of RNA transcripts. Out-of-range values are in bold.

Group	Case No.	Classification	Sex	Age (Years)	TSH	FT4	FT3	TPO-Ab	Tg-Ab	TR-Ab
No evidence of thyroid autoimmunity	T15	Ctrl	F	62	0.63	13.5	7.5	34	19	0.09
	T16	Ctrl	F	55	0.91	17.3	6.2	34	19	0.09
	T18	Ctrl	F	62	0.95	14.3	6.7	13	19	0.09
	T24	Ctrl	F	59	2.4	12.5	5.7	34	19	0.09
Autoimmune thyroid disorders	T21	GD	F	69	**0.029**	15.7	**8.1**	**1280**	**422**	**12**
	T23	GD	F	64	**0.029**	**25.3**	6.3	**429**	**269**	**88**
	T131	GD	F	38	**0.029**	**31.1**	**17.1**	**106**	19	**32.6**
	T148	HT	F	41	1.23	14.4	5.2	**927**	19	0.09

Ctrl, control; GD, Graves’ disease; HT, Hashimoto’s thyroiditis; TSH, thyroid-stimulating hormone (mU/L, range, 0.5–3.6); FT4, free thyroxin (pmol/L, range, 8–20); FT3, free triiodothyronine (pmol/L, range, 3.5–7.5); TPO-Ab, thyroid peroxidase autoantibodies (IU/mL, ref. < 35); Tg-Ab, thyroglobulin autoantibodies (IU/mL, ref. < 20); TR-Ab, TSH-receptor autoantibodies (IU/L, ref. < 1.8). In bold are values outside normal range.

**Table 2:** Partial RNA genome sequences of enterovirus strains obtained from thyroid tissue of patients with autoimmune thyroid disorders.

Case No.	Representative Sequence	Genome Region	Best Matching EV Species	Identities	Gaps
T21	TAATTGGTAGTCCTCCGGCCCCTGAATGCGGCTAATCCTAACTGCGGAGCAGACACCCACGATCCAGTGGGCAGTCTGTCGTAATGGGAAACTCTGCAGCGGAACCGACTACTTTGGGTGTCCGTGTTTCCTTTTATTCTTATACTGGCTGCTTATGGTGACAATCA	5′ UTR	B (echovirus/enterovirus B)	161/165	0
T23	TGAATGCGGGCAGACACCCACGTCCAGTGGGCAGTCTGTCGTAATGGGAACTCTGCAGCGGAACCGACTACTTTGGTGTACCGTGTTTCA	5′ UTR	B (echovirus/EV-B)	83/89	5
T131	TCGTCCGTTCCCACAGTTGCCCGTTACGACTATTCCACATGGTGGCTTCCATGCAATTTTTCTGTGGGGTAGGATTATCCCGCATTCAGGGGCCGGAGGAAG	5′ UTR	Rhinovirus C	88/97	5
T148	TAACTGCAGAGCACATGCCCTCAATCCAGGGGGTGGTGTGTCGTAATGGGCAACtCTGCAGCGGAACCGACTACTTTGGTGtCCGTGTTTCAAT	5′ UTR	A (coxsackievirus A6)	90/92	1

**Table 3:** Gene changes in type I interferon pathways. AV3 cells incubated with enterovirus strains obtained from cases of autoimmune thyroid disorders vs. AV3 cells incubated with a virus-free medium from controls without evidence of thyroid autoimmunity.

Gene	Gene Function	Log_2_ Fold Change	Adjusted *p*-Value	Up-/Down- Regulation
Type I IFN induction pathway (response to viral RNA)				
*IFIH1* (MDA5)	Cytoplasmic sensor of viral nucleic acids. Major role in sensing viral infection and in activating the cascade of antiviral responses including the induction of type I IFNs and proinflammatory cytokines.	−0.19	0.436	-
*TLR3*	Key component of innate and adaptive immunity, a nucleotide-sensing TLR activated by dsRNA. Acts via the adapter TICAM1.	−0.05	0.849	-
*TICAM1*	Component of a multi-helicase–TICAM1 complex acting as a cytoplasmic sensor of viral dsRNA. Activates a cascade of antiviral responses, including proinflammatory cytokines.	−0.18	0.144	-
*TBK1*	Following activation of toll-like receptors by viral or bacterial components, is associated with TRAF3 and TANK and phosphorylates IFN regulatory factors IRF3 and IRF7 and DDX3X. This activity allows subsequent nuclear translocation of IRFs leading to transcriptional activation of type I IFNs and proinflammatory cytokines. Activates IRF3 by phosphorylating innate adapters MAVS, TMEM173/STING, TICAM1, thus leading to recruitment of IRF3.	−0.10	0.504	-
*IFI16*	After binding to viral DNA in the cytoplasm recruits TMEM173/STING and mediates the induction of IFN-beta. Has anti-inflammatory activity and inhibits activation of the AIM2 inflammasome	−0.33	0.032	↓
*TMEM173* (STING1)	Facilitator of innate immune signaling that acts as a sensor of cytosolic DNA from viruses and bacteria and promotes the production of type I IFN.	−0.19	0.054	-
*IRAK1*	IL-1 receptor associated kinase 1 phosphorylates interferon regulatory factor 7 (IRF7) to induce its activation and translocation to the nucleus, resulting in transcriptional activation of type I IFN genes.	−0.26	0.010	↓
*TRAF1*	Adapter molecule that regulates activation of NFKB and JNK.	−0.29	0.144	-
*TRAF2*	Regulates activation of NFKB and JNK. Regulates cell survival and apoptosis.	−0.29	0.015	↓
*TRAF3*	Regulates pathways leading to the activation of NFKB and MAP kinases. Regulates B cell survival. Part of signaling pathways leading to production of IFN and cytokines. Role in T cell-dependent immune responses.	−0.01	0.953	-
*TRAF4*	Activation of NFKB and JNK in response to signaling through TLRs. Regulates cell survival and apoptosis.	−0.25	0.017	↓
*TRAF5*	Mediates activation of NFKB.	−0.62	0.005	↓
*TRAF6*	Activation of NFKB and JUN. Role in dendritic cells maturation and/or activation.	−0.22	0.022	↓
*IKBKB* (IKKB)	Ikappa kinase (IKK) is an enzyme complex that is part of the NFKB signaling pathway. The IKK complex is comprised of three subunits alpha, beta and gamma. Alpha and beta subunits are catalytically active whereas the gamma subunit has regulatory functions.	−0.53	0.002	↓
*IKBKG* (NEMO)		−0.26	0.019	↓
*IKBKE* (IKKE)	Noncanonical IKB kinase (IKK) essential for regulating antiviral signaling pathways.	−0.11	0.528	-
*NFKB1*	NFKB is a homo- or heterodimeric complex formed by the Rel-like domain-containing proteins RELA/p65, RELB, NFKB1/p105, NFKB1/p50, REL and NFKB2/p52 and the heterodimeric p65–p50 complex. Dimers bind at kappa-B sites in the DNA of target genes and individual dimers have distinct preferences for different kappa-B sites.	0.39	0.004	↑
*NFKB2*	NFKB2 has dual functions such as cytoplasmic retention of attached NFKB proteins by p100 and generation of p52 by cotranslational processing.	−0.41	0.003	↓
*NFKBIA*	Member of the NFKB inhibitor family. The protein interacts with REL dimers to inhibit NFKB/REL complexes which are involved in inflammatory responses.	−0.04	0.690	-
*NFKBIZ*	Member of the ankyrin repeat family of proteins known to play a role in inflammatory responses. Activates IL-6 but decreases TNF-alpha production.	−1.16	<0.001	↓
*RELA*	The NF-kappa-B heterodimeric RELA–NFKB1 and RELA–REL complexes function as transcriptional activators. The NFKB homodimeric RELA–RELA complex activates IL-8 expression.	0.46	0.040	↑
*RELB*	NFKB heterodimeric RelB–p50 and RelB–p52 complexes are transcriptional activators. RELB is required for both T and B lymphocyte maturation and function.	0.18	0.171	-
*IRF1*	Transcriptional regulator serving as an activator of genes involved in antiviral response, including as type I IFNs IFN-alpha/beta, DDX58/RIG-I, TNFSF10/TRAIL, OAS1/2, PIAS1/GBP, EIF2AK2/PKR and RSAD2/viperin.	−0.74	0.002	↓
*IRF3*	Key transcriptional regulator of type I IFN-dependent immune responses against viruses. Regulates transcription of type I IFN genes and IFN-stimulated genes by binding to an IFN-stimulated response element (ISRE).	−0.03	0.825	-
*IFITM1*	IFN-induced antiviral protein which inhibits the entry of viruses to the cytoplasm permitting endocytosis but preventing subsequent viral fusion and release of viral contents into the cytosol.	−0.44	0.007	↓
Type I IFN signaling pathway (response to IFN)				
*IFNAR1*	Heterodimer with IFNAR2. Type I IFN binding activates the JAK–STAT signaling cascade and triggers tyrosine phosphorylation of proteins including JAKs, TYK2, STAT and the IFNR alpha- and beta-subunits themselves.	0.37	0.041	↑
*IFNAR2*	Associates with IFNAR1 to form the type I IFN receptor. Involved in IFN-mediated STAT1, STAT2 and STAT3 activation. Isoforms 1 and 2 are involved in signal transduction due to their association with JAK1.	0.15	0.081	-
*JAK1*	Tyrosine kinase of the non-receptor type involved in the IFN-alpha/beta/gamma signal pathway.	0.42	0.022	↑
*JAK2*	Tyrosine kinase of the non-receptor type involved in different processes (cell growth, differentiation, histone modifications). Mediates signaling events in both innate and adaptive immunity. In the cytoplasm, mediates signal transduction via association with type II receptors (IFN-alpha, IFN-beta, IFN-gamma and multiple interleukins).	−0.32	0.036	↓
*TYK2*	Tyrosine kinase that is associated with the cytoplasmic domain of type I and type II cytokine receptors and promulgates cytokine signals by phosphorylating receptor subunits. Component of type I and type III IFN signaling pathways.	−0.41	0.006	↓
*TBK1*	TANK binding kinase 1. TBK1 plays a key role in IRF3 activation. First phosphorylates innate adapter proteins MAVS, STING1, TICAM1 leading to recruitment and phosphorylation of IRF3. Phosphorylated IRF3 enters the nucleus to induce expression of interferons.	−0.10	0.504	-
*STAT1*	Transcription activator that mediates cellular responses to IFNs, cytokines, growth factors. Following type I IFN binding to cell receptors, signaling via protein kinases leads to activation of TYK2 and JAK1 and to tyrosine phosphorylation of STAT1 and STAT2. Phosphorylated STATs are associated with ISGF3G/IRF-9, the ISGF3 complex transcription factor that enters the nucleus and promotes transcription of IFN-stimulated genes which drive the cell in an antiviral state.	0.66	0.011	↑
*STAT2*	Signal transducer and activator of transcription that mediates signaling by type I IFNs.	−0.49	0.007	↓

**Table 4:** Differentially expressed cytokine-related transcripts. AV3 cells incubated with enterovirus strains obtained from cases of autoimmune thyroid disorders vs. AV3 cells incubated with a virus-free medium from controls without evidence of thyroid autoimmunity.

Gene	Gene Function	Log_2_ Fold Change	Adjusted *p*-Value	Up-/Down- Regulation
*EGR1*	Early growth response 1. Transcriptional regulator of cytokines such as IL-1B and CXCL2 that are involved in inflammatory processes.	−1.25	0.002	↓
*CXCL2*	Chemoattractant active on T lymphocytes and monocytes but not on neutrophils. Activates the C–X–C chemokine receptor CXCR4 to induce an increase in intracellular calcium ions and chemotaxis.	−1.14	<0.001	↓
*EGR2*	Early growth response gene 2. Regulatory molecule suppressing excessive immune responses.	−0.95	0.004	↓
*CEBPB*	CCAAT-enhancer-binding-protein beta. Binds to regulatory regions of several acute-phase and cytokine genes and plays a role in the regulation of acute-phase reactions and inflammation.	−0.89	<0.001	↓
*SOCS3*	STAT-induced STAT inhibitor, suppressor of cytokine signaling. The product of this gene is induced by different cytokines and inhibits the JAK2 kinase.	−0.88	<0.001	↓
*LIF*	Cytokine of the IL-6 family. Regulates hematopoietic differentiation and neuronal cell differentiation, stimulates synthesis of acute-phase proteins in hepatocytes.	−0.62	0.003	↓
*PDGFB*	Platelet-derived growth factor beta: regulation of cell proliferation, cell migration, survival and chemotaxis. Inhibitor of inflammatory responses, mitogen for cells of mesenchymal origin.	−0.58	0.002	↓
*IL6*	Functions in inflammation and maturation of B cells. Produced at sites of acute and chronic inflammation. Endogenous pyrogen in autoimmune diseases and infections. Transcriptional inflammatory response through interleukin 6 receptor-alpha.	−0.50	0.050	↓
*IL20*	Related to IL-10, transduces its signal through STAT3. Related pathways are ERK signaling and TGF-beta pathways.	−0.37	0.025	↓
*TGFB1*	Binds TGF-beta receptors activating the SMAD family transcription factors. The active form consists of a mature peptide homodimer that may form heterodimers with other TGF-beta family members. Regulates cell proliferation, differentiation, growth and expression of other factors including IFN-gamma and TNF-alpha.	−0.36	0.005	↓
*IL1B*	Produced by activated macrophages as proprotein that is proteolytically processed to its active form by caspase 1 (CASP1/ICE). It is an important mediator of the inflammatory response and is involved in cell proliferation, differentiation, apoptosis.	−0.34	0.007	↓
*GPI*	Glucose phosphate isomerase protein family. Within the cytoplasm, functions as a glycolytic enzyme that interconverts glucose-6-phosphate and fructose-6-phosphate. Extracellularly, GPI is also referred to as a neuroleukin and functions as a neurotrophic factor and as a lymphokine that induces immunoglobulin secretion.	−0.28	0.008	↓
*CCL2* (MCP1)	Chemotactic activity for myeloid and lymphoid cells, not for neutrophils or eosinophils. Binds to chemokine receptors CCR2 and CCR4. Implicated in diseases characterized by monocytic infiltrates.	0.23	0.049	↑
*IL18*	Proinflammatory cytokine; augments NK cell activity in spleen cells, stimulates IFN-gamma production in T helper cells.	0.31	0.032	↑

**Table 5:** Significantly deregulated pathways. AV3 cells incubated with enterovirus strains obtained from cases of autoimmune thyroid disorders vs. AV3 cells incubated with virus-free control supernatants. In bold are significantly deregulated pathways.

Pathway	Statistical Mean ^1^	*p*-Value	Number of Genes in the Pathway
**hsa04722, neurotrophin signaling pathway**	**0.94**	**0.032**	**19**
**hsa04620, toll-like receptor signaling pathway**	**0.88**	**0.040**	**29**
**hsa04660, T cell receptor signaling pathway**	**0.88**	**0.042**	**18**
hsa04380, osteoclast differentiation	0.82	0.051	32
hsa04650, natural killer cell-mediated cytotoxicity	0.80	0.058	19
hsa04662, B cell receptor signaling pathway	0.73	0.075	19
hsa04810, regulation of actin cytoskeleton	0.71	0.082	12
hsa04622, RIG-I-like receptor signaling pathway	0.68	0.090	20
hsa04370, VEGF signaling pathway	0.65	0.104	11

^1^ Quantitative estimate of pathway deregulation.

## Data Availability

The data presented in this study are available on request from the corresponding author.

## Supplementary Materials

The following are available online at https://www.mdpi.com/article/10.3390/microorganisms9040876/s1, Table S1: List of consumables, chemicals, cell lines, viruses, culture media, antibodies, reagents for molecular biology, commercial kits, instruments, applications and software; Table S2: List of differentially expressed gene transcripts in AV3 cells incubated with enterovirus strains obtained from cases of autoimmune thyroid disorders vs. AV3 cells incubated with a virus-free medium from controls without evidence of thyroid autoimmunity.
